# Alveolar Bone Repair Dynamics in Rats Influenced by Coffee Ingestion: A Confocal Microscopy Analysis

**DOI:** 10.7759/cureus.79702

**Published:** 2025-02-26

**Authors:** Kaio dos Santos, Alexandre R Freire, Beatriz C Ferreira-Pileggi, Isabella Andreazza de Freitas, Roberta Okamoto, Felippe B Prado, Ana Cláudia Rossi

**Affiliations:** 1 Biosciences, Piracicaba Dental School-University of Campinas (UNICAMP), Piracicaba, BRA; 2 Medicine, Botucatu Medical School, São Paulo State University Júlio de Mesquita Filho-Faculty of Medicine of Botucatu (FMB), Botucatu, BRA; 3 Basic Sciences, Araçatuba Dental School-Paulista State University (UNESP), Araçatuba, BRA

**Keywords:** alveolar bone, coffee, fluorochromes, rat, tooth extraction

## Abstract

After surgical tooth extraction, it is necessary to maintain the environment of the oral cavity in appropriate conditions to favor alveolar bone repair. Coffee includes multiple bioactive compounds; some of these compounds can affect bone metabolism. The aim of this study was to investigate the effect of coffee ingestion on alveolar bone dynamics after tooth extraction using confocal laser microscopy. Eight male Wistar rats, aged two months, were used and divided into two groups: the control group, in which the normal diet was maintained, and the experimental group, with coffee ingestion. In both groups, extraction of the upper right incisor was performed. The fluorochromes calcein was injected at 14 days and alizarin at 28 days in rats. The sample was euthanized 28 days after the tooth extraction. The right maxilla was removed, processed, and analyzed under a confocal laser microscope, and the mineral apposition rate and the alveolar bone area marked with calcein and alizarin were obtained. There was no statistically significant difference between groups for bone mineralization rate. The two-way ANOVA compared differences between groups and fluorochromes; the interaction between the groups was not statistically significant. The intragroup analysis showed a statistically significant difference between the fluorochromes injected at 14 days and 28 days after tooth extraction. There was a tendency for there to be a decrease in renewed bone in the group that drank coffee, although this was not significant.

## Introduction

Alveolar bone repair is a dynamic process that involves a series of tissue reactions that occur within the alveolus after tooth extraction, with the objective of filling the dental alveolus with bone tissue [1-3]. The process of alveolar bone healing in rats is fairly established in the literature [4]. Preclinical studies have analyzed the alveolar bone after extraction under clinical and experimental conditions, and it is known that its process is completed 28 days after the day of tooth extraction [1, 5]. Só et al. [4] reported that, when it comes to alveolar bone healing analysis, rats are the most frequently used animal model, and the upper incisors and maxillary molars are the most frequently extracted teeth. A classic model is the extraction of upper incisors in rats, describing alveolar healing in three phases: clot formation and cell proliferation from the connective tissue; connective tissue formation and scarring; and ossification phase [1].

After surgical tooth extraction, it is necessary to maintain the environment of the oral cavity in appropriate conditions to favor alveolar bone repair. Healing and tissue repair have factors that can positively or negatively affect this process. The potential for bone healing is influenced by several mechanisms and factors, such as aging, hormonal, biochemical, and biomechanical ones [6-7]. Moreover, the diet and its components act as a critical component in all wound healing processes, as they can exert influence over ideal conditions to favor alveolar bone repair or not [8-9]. This happens through the management of the behavior and function of the cells accountable for the formation of new bone [6].

Coffee includes multiple bioactive compounds, some with potentially therapeutic antioxidant, anti-inflammatory, antifibrotic, or anticancer effects [10-12]. Some of these compounds can affect bone metabolism [12]. Substances, such as antioxidants, may provide bone protection, while other specific substances may increase bone resorption [12-13]. Despite research into this field, there is inconsistency in the association between coffee consumption and musculoskeletal outcomes, and the impact of coffee consumption on bone metabolism remains controversial [11-12].

Regarding alveolar bone repair, there is a study carried out in 2015 that showed the daily intake of boiled coffee and the administration of pure caffeine affected the bone repair process after tooth extraction in rats, including a delay in the production of granulation tissue [14]. However, there are no studies showing the impact of daily coffee consumption on alveolar bone osteogenesis.

Fluorochrome markers have already been used in studies with rats to detect and measure osteogenic activities [15-16]. Fluorochromes are chemical compounds that can bind calcium at the time of precipitation in the organic bone matrix. Therefore, the fluorochrome markings represent the amount of calcium precipitation, thus allowing the measurement of bone formation [17]. This marker allows us to assess a possible alteration caused by coffee at the time of osteogenesis in alveolar repair.

In the present study, through fluorochrome markers, it was possible to analyze whether there is any impact on post-repair osteogenesis, since clinical studies present conflicting results in relation to calcium metabolism, and this mineral is directly linked to bone matrix mineralization. Thus, it is expected to contribute to the dental field through knowledge of the influence of coffee on alveolar bone repair after tooth extraction, allowing the targeting of treatments, for example, with dental implants, in which the presence of a healthy bone and biomechanically active is required. The aim of this study was to investigate the effect of coffee ingestion on mineral apposition rate and bone area during alveolar bone repair after tooth extraction in rats to clarify its potential influence on bone regeneration processes relevant to dental health, using confocal laser microscopy.

## Materials and methods

Experimental design

This study was approved by the Ethics Committee on Animal Experimentation (CEUA) of the Institute of Biology, University of Campinas (UNICAMP). All procedures in this work were performed according to national legislation enforced by the National Council of Animal Experimentation Control (CONCEA) of Brazil under protocol number 5324-1/2019.

Eight male Wistar rats (*Rattus norvegicus albinus*), two months old and weighing between 200 and 250 g, were used. The animals in the control group were kept in collective cages (four animals/box), and the animals in the group with coffee ingestion were kept in individual cages, with a temperature of 22 ± 2ºC, a controlled light cycle (12/12 hours), and free access to water and feed until two months of age. After this period, the control group continued to have free access to water and feed, but the group with coffee ingestion had controlled access to water and free feed.

**Figure 1:**
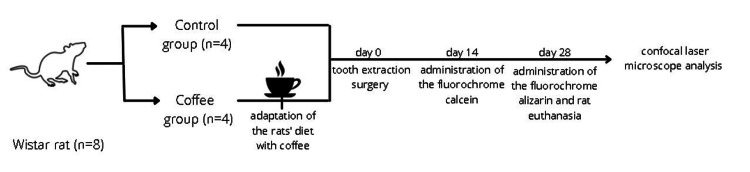
Chronological flowchart of events in this study

The rats were randomly distributed into two groups for the experiments: A) Control group: the rats received free access to water before and after tooth extraction surgery (n=4); B) Coffee group: the rats received free access to water before tooth extraction surgery; the rats received water and coffee after tooth extraction surgery (n=4);

Figure 1 shows the sequence of the events in the present study.

Adaptation model to the introduction of coffee

The amount of coffee ingested by the animals was estimated based on the daily human consumption of four cups (240 mL) per day for a person weighing 60 kg [14]. Thus, the rats started to receive roasted, ground, and cooked coffee (Morro Grande Coffee®, Piracicaba, São Paulo, Brazil) for the adaptation of 50 mg/mL (1.2 mL of coffee infusion/day), reducing the supply of water during the 28 days from day 0 of tooth extraction. The rats of the coffee group were placed in cages, separately (one per cage), to control the coffee ingestion.

The animals in the control group were kept in collective cages (four animals/box), and the animals in the group with coffee ingestion were kept in individual cages, with a temperature of 22 ± 2ºC, a controlled light cycle (12/12 hours), and free access to water and feed until two months of age. The relative humidity was between 40% and 60%. After this period, the control group continued to have free access to water and feed, but the group with coffee ingestion had controlled access to water and free feed. The rats received the special Purina® rat food (Société des Produits Nestlé S.A., Vevey, Switzerland).

Tooth extraction surgery

The tooth extraction surgery was performed according to the technique of Okamoto and Russo [18]. The procedure was performed under sedation via an intraperitoneal injection of ketamine (40-87 mg/kg; ketamine chloride, injectable, Fort Dodge Saude Animal Ltda., São Paulo, Brazil) to promote anesthesia and xylazine (5-13 mg/kg) (Xilazine-Coopers, Brazil, Ltda) to promote muscle relaxation. Then, antisepsis with polyvinylpyrrolidone iodide (Indústria Química e Farmacêutica Rioquímica Ltda, São Paulo, Brazil) was performed, and the right upper incisor was extracted using specific and adapted instruments [18]. The gingival mucosa was sutured with polyglactin 910 (Vicryl 4.0, Johnson & Johnson, New Brunswick, NJ, USA). After the surgery procedure, promote analgesia using an injection of ketoprofen (non-steroidal anti-inflammatory drug (NSAID), 5 mg/kg) subcutaneously, once a day.

Fluorochrome injections

In both control and coffee groups, 2 µg/kg body weight of intravenous green calcein was administered (Sigma Chemical Co., St. Louis, MO, USA) 14 days from the day of the extraction was performed. Calcein was prepared immediately before use. Each animal received doses of 1 mL of dye volume. In both groups, 2.5 µg/kg body weight of intravenous alizarin red was administered (Sigma Chemical Co.) 24 days from the day the extraction was performed. Alizarin was prepared immediately before use. Each animal received doses of 1 mL of dye volume.

Euthanasia

The rats from both groups were euthanized 28 days after day 0 from tooth extraction surgery using an excessive anesthetic dose. The head was disjointed from the body and dissected to obtain the skull and fixed in 10% formalin solution and 0.1M phosphate buffer (pH 7.4) for 24h at 4°C.

Laboratory procedures

Using an increasing sequence of alcohols (70% to 100%), the dissected maxilla was dehydrated. After the dehydration process, the solutions were soaked in methyl methacrylate (MMAL) solutions (Classic, Classic Dental Articles, São Paulo, Brazil) in three baths to fix the pieces. The catalyst used was benzoyl peroxide (1%, Riedel-de Haën AG, Seelze, Hannover, Germany) added only in the last bath [2]. To obtain complete polymerization, the pieces were kept in test tubes inside an oven at 37º C for five days. After polymerization, the resin blocks were cut in the sagittal plane with the aid of a Maxicut mounted on a bench motor (Kota, São Paulo, Brazil). The parts were then rubbed bilaterally with 120, 300, 400, 600, 800, and 1200 crescent grains mounted in an automatic polisher (ECOMET 250PRO / AUTOMET 250, Buehler, Lake Bluff, IL, USA) until the cuts reached a thickness of 80 µm. For the measurement, a digital caliper was used (Mitutoyo, Pompeia, Brazil). The slices were mounted in glass and mineral oil slides (liquid oil, Mantecor, Taquara, Brazil) and sealed with a glass lid and enamel transparent to prevent oil leakage and possible dehydration of the samples [17].

Confocal laser microscopy

Longitudinal sections of the area of interest (bone adjacent to the apical third of the right upper incisor) were obtained using a Leica TCS SP5 microscope (Leica Microsystems, Heidelberg, Germany) through a 10x objective (original magnification 100x). Thus, the area of the bone around this tooth was evaluated in each specimen.

In the microscope configuration, the laser excitation with 488nm and emission ranging from 500 to 569nm were used for the calcein. For alizarin, the laser excitation with 405 nm and emission ranging from 413 to 473nm were used, which allowed the visualization of the fluorochromes.

The images obtained showed two colors representing calcium precipitation after the administration of calcein (green) and alizarin (red) at 28 days after extraction. The software overlays the images and presents both overlapping fluorochromes. The prominence of green color represented a higher amount of an older bone, whereas a red exaltation represented a higher amount of a younger bone [2, 17].

Bone area analysis

These images were saved in tagged image file format (TIFF) format and transported to ImageJ software (National Institutes of Health, Bethesda, ML, USA and the Laboratory for Optical and Computational Instrumentation (LOCI, University of Wisconsin, Madison, WI, USA)). Using the known scale of each slide, the software measurements were calibrated. With the "color threshold" tool, each image was standardized according to hue, saturation, and brightness to reveal fluorochromes. The principle, the "free hands" tool, was selected; the calcein was highlighted; and the "measure" tool was used to calculate the bone area in μm². The same procedure was performed with alizarin, with data related to the dynamics of the bone tissue in the region being obtained [2, 17].

Mineral apposition rate

To obtain the mineral apposition rate of the fluorochrome label, images containing the alizarin and calcein overlay were imported into Image J software. Choosing five regions randomly in each image, the "straight" tool was used, and a line was drawn from the beginning of alizarin precipitation (red label) to the end of calcein precipitation (green label). The mean values were divided by 14, representing the period between both fluorochrome injections, leading to the mineral apposition rate value [2, 19].

Statistical analysis

Data from the mineral apposition rate were evaluated using the Mann-Whitney test (two-tailed). Data from the bone area were analyzed with a two-way ANOVA. Significant results were statistically evaluated with Sidak's multiple comparisons test. All tests showed a significance level of 5%. The statistical program GraphPad Prism v.8 (La Jolla, CA, USA) was used.

## Results

**Figure 2:**
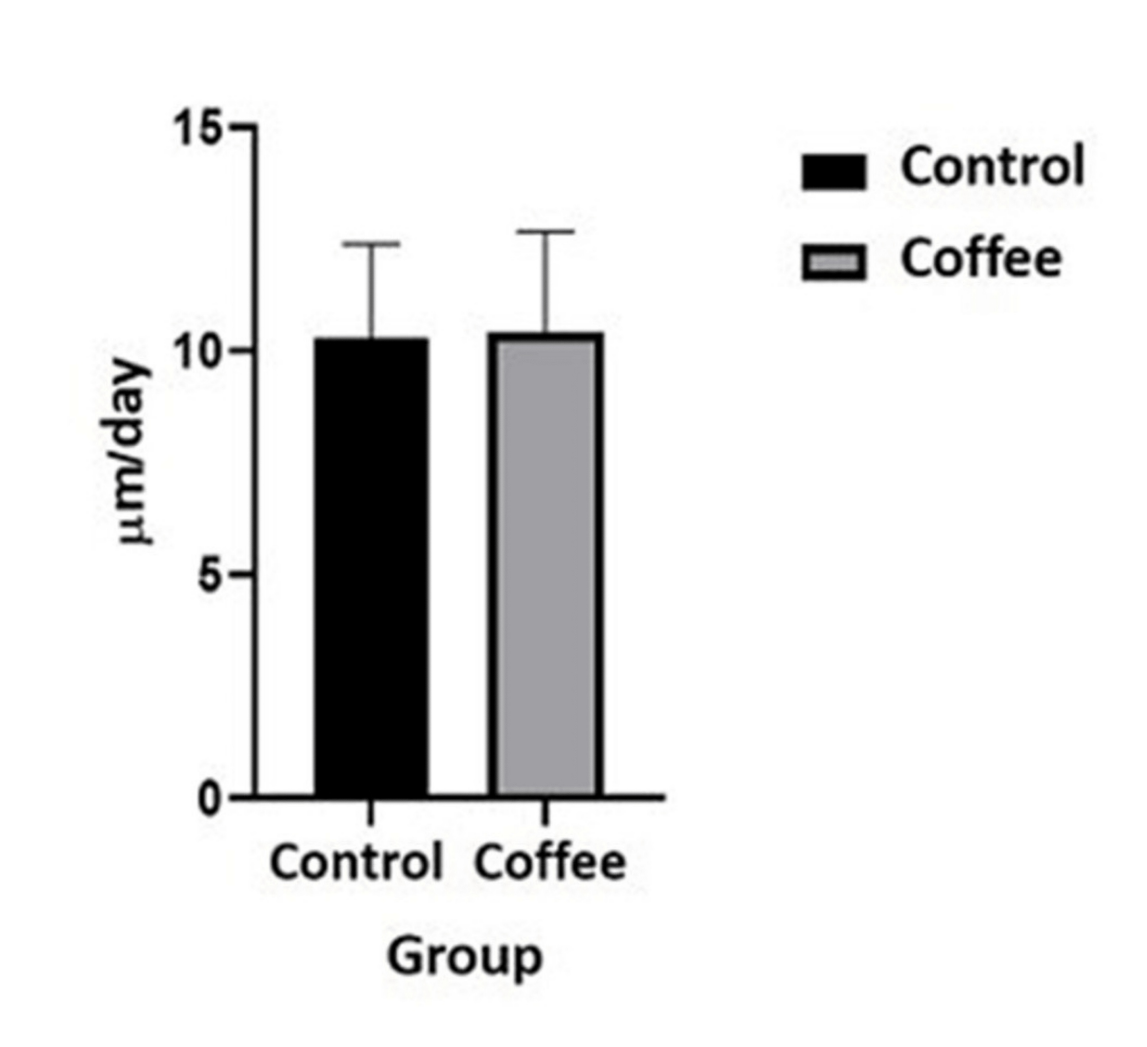
The mineral apposition rate per day (μm) of the coffee and control groups was evaluated linearly. Results are represented by the mean and error bars by SEM. SEM: standard error of the mean

There was no statistically significant difference between groups for mineral apposition rate (Mann Whitney test, P=0.7756) (Figure 2).

**Figure 3:**
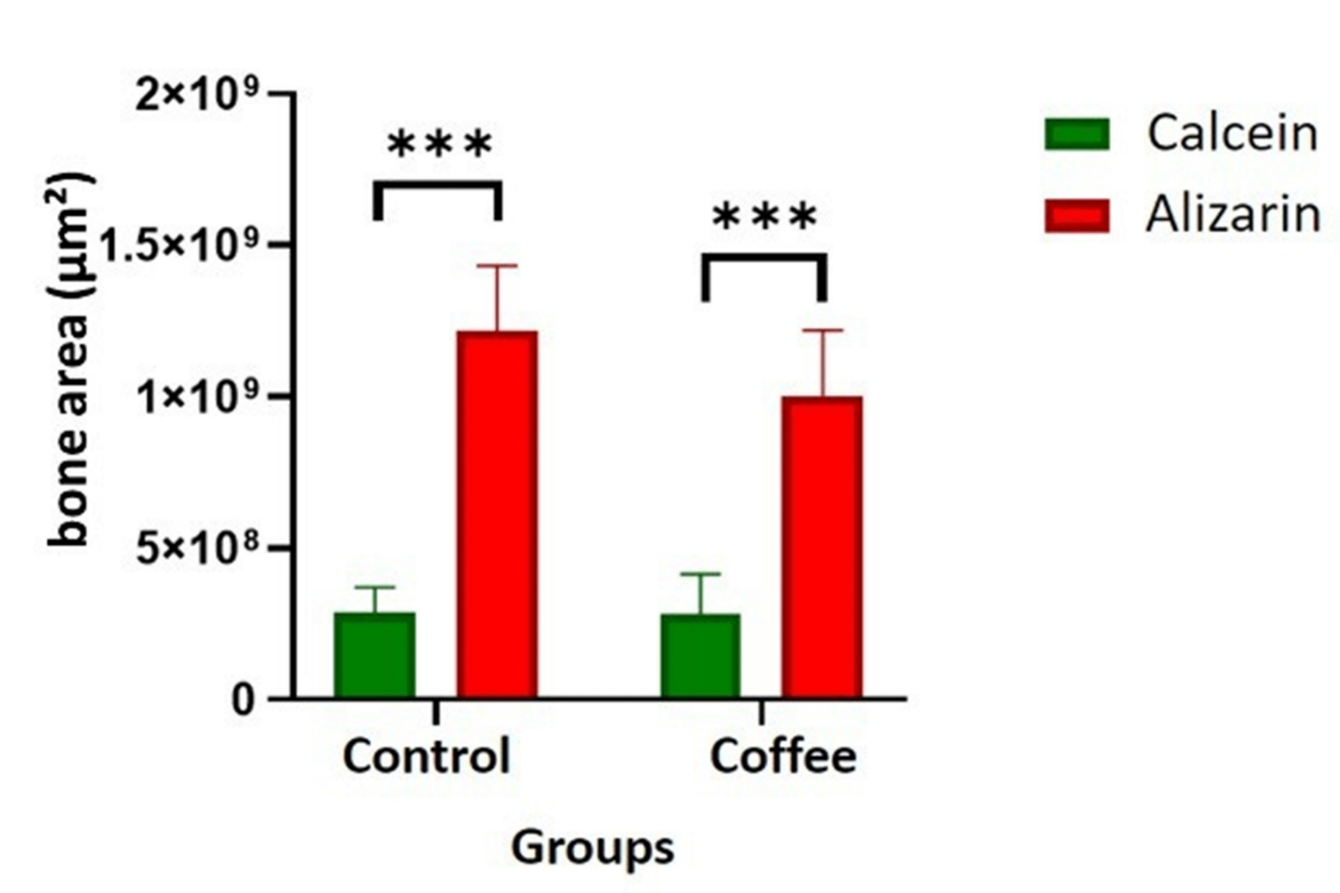
Bone area (μm²) for the experimental group (coffee) as assessed by calcein and alizarin staining. *** Tukey test P<0.0001 (intragroup comparisons of statistical significance).

Data were analyzed with two-way ANOVA to compare differences between groups (control and coffee) and fluorochromes (calcein and alizarin). The interaction between the groups was not statistically significant. The interaction between the fluorochromes was statistically significant (P<0.0001). The intragroup analysis showed a statistically significant difference between the fluorochromes injected at 14 days (calcein) and at 28 days (alizarin) after tooth extraction (Tukey test P<0.0001) (Figure 3). In the coffee group, there was a non-significant trend to have a decrease in alizarin.

**Figure 4:**
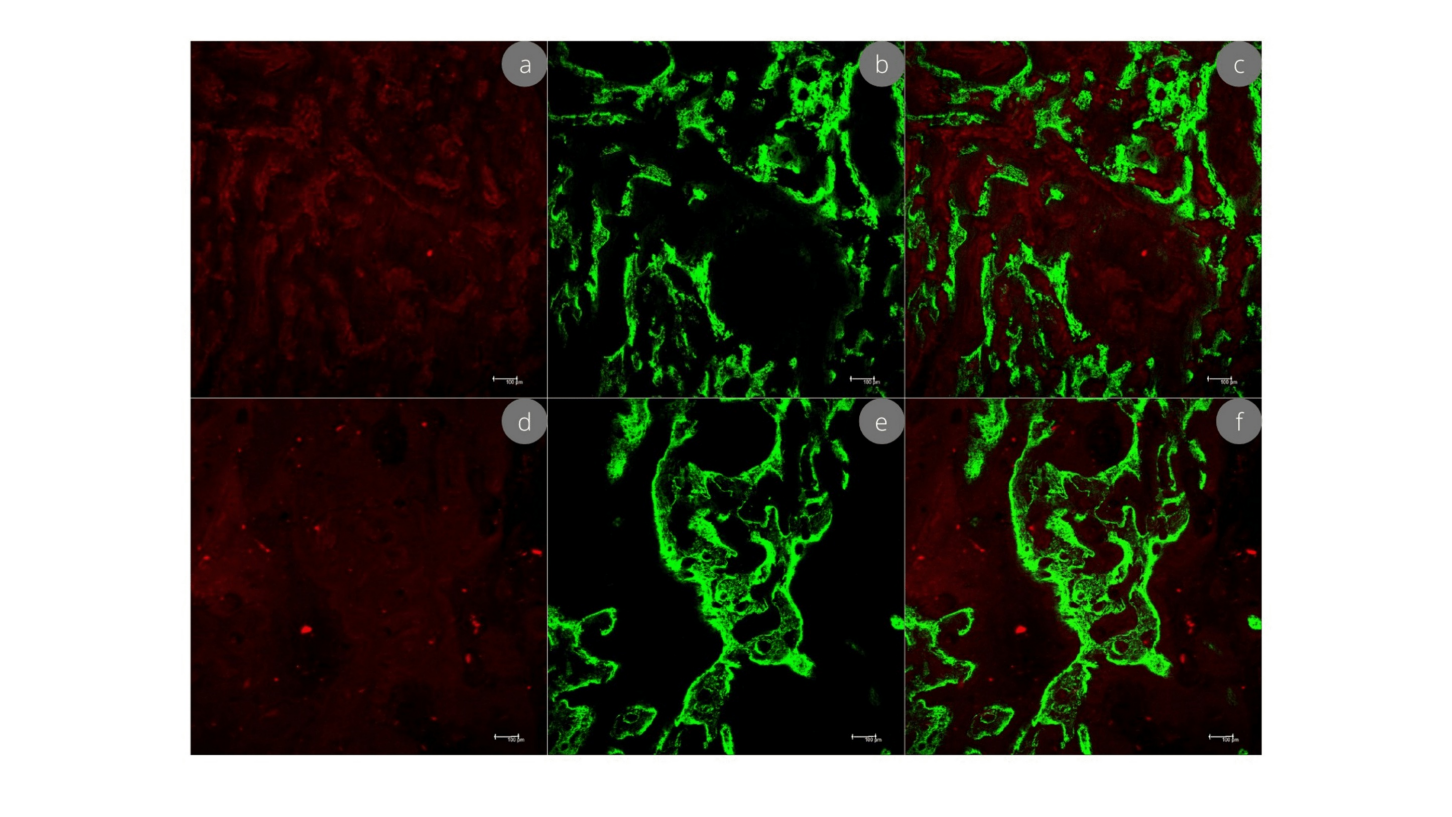
Alveolar bone images obtained using confocal microscopy a, b, and c are the bone area of the control group; d, e, and f are the bone area of the coffee group; a and d represent calcium precipitation after the administration of alizarin (red) at 28 days after extraction; b and e represent calcium precipitation after the administration of calcein (green) at 28 days after extraction; c and f present both overlapping fluorochromes.; scale bar: 100 micrometers.

Figure 4 shows the image of the amount of old bone and new bone. Green color (calcein) marks the older bone, whereas red (alizarin) marks the new bone.

## Discussion

The impact and relevance of coffee on health are based on the knowledge that it is a beverage that is consumed all over the world. It was described that coffee is the drink with the largest proportion of adults reporting consumption, after water [20]. It includes a wide array of components that can have potential implications on people’s health [10, 11].

A study conducted in the Brazilian population showed that, between the years 2008 and 2009, the estimated average daily daily coffee intake was 163 mL [20]. The Brazilian Institute of Geography and Statistics (IBGE) communicated that coffee is the most daily consumed food by 79% of the Brazilian population over 10 years of age [21]. The Coffee Consumption Trends survey carried out in 2010 showed that 97% of respondents are coffee consumers, and the main reason reported for the consumption of the beverage among the participants was the fact that it is a family habit or tradition, brought since childhood, in addition to having characteristics associated with pleasure and sociability [22].

Previous work [23] showed that growing rats that had caffeine in their diet had decreased mineralization of the bone matrix of their tibias. Caffeine caused significant reductions in extracellular matrix production, mineralization, and alkaline phosphatase activity, accompanied by decreased gene expression of cartilage-specific matrix proteins such as aggrecan, type II, and type X collagen. Studies have also shown the detrimental effects of caffeine on bone tissue, impacting calcium balance [24-27].

Fluorochromes have the property of binding to calcium at the moment that it is going to precipitate on the organic bone matrix. Thus, because of this direct relation, the extent of fluorochrome labeling represents the quantity of calcium precipitation and consequently quantifies the bone formation event [17, 28-29]. To obtain the mineral apposition rate in the current paper, two fluorochromes (calcein and alizarin) were injected in the rats from both the control and coffee groups. At 14 days from the day of the tooth extraction, intravenous green calcein was administered, and, at 24 days from the day of the tooth extraction, intravenous alizarin red was administered. The first fluorochrome injected, calcein, indicates calcium deposition in old bone. The last injected fluorochrome, alizarin, represents the newly formed bone, which can be called the active surface of mineralization [16-17].

In the present study, there was no statistically significant difference between groups for mineral apposition rate. Despite that, the coffee group showed a non-significant trend to have a decrease in alizarin, i.e., the young bone is reduced. This event was explained by Zhou et al. [30], who described that the caffeine contained in coffee causes a reduction in the differentiation of mesenchymal stem cells in osteogenic lineages and inhibits some specific gene expression, thus affecting osteogenesis. It is assumed that caffeine participates in the expression regulation of the Cbfa1/Runx2 gene and decreases the differentiation rate of mesenchymal stem cells in osteoblasts [12]. Samoggia and Riedel [31] reported that compounds of coffee, notably caffeine, weaken calcium absorption and promote its excretion [31].

In the present study, the administration of fluorochromes formed fluorescent lines by calcium precipitation in the organic matrix next to calcein (Green fluorochrome given on day 14) and by calcium precipitation next to alizarin (red fluorochrome given on day 28). Then, the green marker on the slides is the mineralization of the bone formed in the middle of the alveolar repair of both groups. As alizarin (red) is the fluorochrome administered at the end of alveolar bone repair, red was observed in the most recent mineralization on the slides, that is, the bone formed at the end of the repair. The decrease of alizarin in the coffee group showed the impact of coffee on calcium metabolism, which generates a non-significant decrease in mineralization in alveolar repair.

Since research has shown that coffee is among the most consumed foods in Brazil [32], the present study figured out the consequences that diet can have on bony metabolic processes and how this affects alveolar bone repair. There are several postoperative recommendations for patients, including not consuming hot drinks for the first few days after surgery. However, after a short time, patients return to consuming their diet normally, including coffee, which can be done in the first few days if this drink is made cold, as it is found in some foods. This consumption then returns even before an alveolar bone repair is completed. In this paper, the effects of coffee ingestion on the alveolar bone dynamics after dental extraction in Wistar rats. Some of the previous studies [14, 33-38] found in the literature aimed to analyze the influence of caffeine on bone. That implies different results, once coffee includes a complex mixture of many bioactive compounds with physiological effects, and caffeine is just one of them. Coffee includes up to 1,000 phytochemicals, such as phenols, chlorogenic and caffeic acid, lactones, and others [10-12]. Another thing that must be considered is that caffeine is found in several foods and medicines consumed by the population. Thus, not only coffee present in the diet but also various products that are consumed can add caffeine, which, with a non-significant trend for less mineralization found in this study, can cause the tissue to be less mineralized after repair. Thus, analyzing the effects of the caffeine itself brings results that are different from those obtained when analyzing the effects of coffee ingestion.

Besides that, the composition of coffee may vary depending on the method of its preparation. The amount of caffeine in a cup of coffee, for example, is influenced by the method of coffee preparation [10]. This can possibly explain why some results found in previous studies and the impact of coffee consumption on bone metabolism remain controversial. This study applied only one type and method of coffee preparation, and, considering the influences of dosage, type, and nature of coffee, it can be characterized as a limitation of this and other studies as well.

## Conclusions

In conclusion, this study presented the decrease in alizarin levels in the coffee group and suggests a potential impact of coffee on calcium metabolism. After alveolar repair, the alveolar bone is important as a support for rehabilitating edentulous patients with implants or dental prostheses. Therefore, it is important to study dietary habits after dental extraction surgery and their possible influences on the quality of alveolar repair.

Despite the limitations presented in the present study, it suggests that further studies should be carried out, including variations in the composition of coffee and other methodologies for alveolar bone repair evaluation. Additionally, other tools applied to image analysis, such as microtomography, could enhance the understanding of the effects of coffee on bone formation and remodeling during alveolar repair.
